# Modeling of sodium currents from mesencephalic trigeminal neurons by system identification and sensitivity analysis

**DOI:** 10.1186/1471-2202-14-S1-P75

**Published:** 2013-07-08

**Authors:** Federico Davoine, Sebastián Curti, Pablo Monzón

**Affiliations:** 1 School of Engineering, Universidad de la República, Montevideo, 11200, Uruguay; 2 School of Medicine, Universidad de la República, Montevideo, 11800, Uruguay

## 

Mesencephalic trigeminal (MesV) cells are sensory neurons involved in the brainstem circuitry that generates and controls orofacial activities [1]. Recently we have showed that these neurons are electrically coupled through somato-somatic Cx36 containing gap junctions, forming small networks of strongly coupled cells [2]. Frequency transfer analysis of these contacts in which coupling strength is estimated by means of frequency modulated sine waves (ZAP), demonstrate that these contacts do not behave as simple low pass filters. In fact, transmission of high frequencies is amplified, indicating that coupling of signals with high frequency content, like action potentials, might be relatively stronger. Moreover, this frequency transfer characteristics rely on active currents of the non-synaptic membrane, particularly on a persistent sodium current (I_NaP_) and an A type potassium current (I_A_), in combination with the passive properties [2]. These characteristics promotes strong and precise synchronization of the activity of coupled cells, providing a mechanism for coincidence detection and lateral excitation among these neurons, possibly with functional consequences for the organization and control of orofacial behaviors.

In an attempt to generate a model of a network of electrically coupled MesV neurons that reproduce these behaviors, as critical as the subthreshold active mechanisms responsible for the frequency selectivity of these contacts (i.e. I_NaP _and I_A _currents), is the waveform of the action potentials of these cells characterized by its high amplitude and short duration, with almost no after hyperpolarization and small interspike intervals. Despite the fact that those subthreshold mechanisms have been thoroughly studied [3,4], kinetic data of membrane currents responsible for spike generation is lacking. We developed a model of the sodium currents based on experimental data from whole cell voltage clamp recordings obtained in slices of the rat brainstem following standard procedures [2].

Based on these experimental results, we have developed a state-space model of sodium currents (transient, persistent and resurgent) [5], that was able to fit our recordings from MesV neurons. Model parameters are voltage-dependent in a non-linear manner. In order to find them, for each voltage step, state-space model was formulated as a linear ordinary equation system. Using System Identification toolbox of Matlab, states were predicted using a linear state estimator. The optimal parameters were found by minimizing the error between the prediction of the open state and the experimental data [6]. Then, we derived the explicit variational equations for the sensitivity of the model with respect of the parameters, solving them with XPP. NEURON simulations of a single compartment MesV neuron model confirmed that the proposed model of sodium currents is able to explain the fast dynamics of action potentials. In fact, modeling results showed that I_NaT _present a voltage dependent fast inactivation process that is the main contributor to the action potential repolarization and hence of is waveform, determining its frequency content of the spikes and its ability to pass through electrical contacts between these cells.
